# Blending Gagne’s Instructional Model with Peyton’s Approach to Design an Introductory Bioinformatics Lesson Plan for Medical Students: Proof-of-Concept Study

**DOI:** 10.2196/11122

**Published:** 2018-10-25

**Authors:** Richa Tambi, Riad Bayoumi, Peter Lansberg, Yajnavalka Banerjee

**Affiliations:** 1 Department of Basic Medical Sciences Mohammed Bin Rashid University of Medicine and Health Sciences Dubai United Arab Emirates; 2 Department of Pediatrics University Medical Center Groningen Groningen Netherlands; 3 University of Dundee Department of Medical Education University of Dundee Dundee United Kingdom

**Keywords:** bioinformatics, Gagne’s instructional model, genetics, lesson plan, medical education, Peyton’s approach, undergraduate medical education

## Abstract

**Background:**

With the rapid integration of genetics into medicine, it has become evident that practicing physicians as well as medical students and clinical researchers need to be updated on the fundamentals of bioinformatics. To achieve this, the following gaps need to be addressed: a lack of defined learning objectives for “Bioinformatics for Medical Practitioner” courses, an absence of a structured lesson plan to disseminate the learning objectives, and no defined step-by-step strategy to teach the essentials of bioinformatics in the medical curriculum.

**Objective:**

The objective of this study was to address these gaps to design a streamlined pedagogical strategy for teaching basics of bioinformatics in the undergraduate medical curriculum.

**Methods:**

The established instructional design strategies employed in medical education—Gagne’s 9 events of instruction—were followed with further contributions from Peyton’s four-step approach to design a structured lesson plan in bioinformatics.

**Results:**

First, we defined the specifics of bioinformatics that a medical student or health care professional should be introduced to use this knowledge in a clinical context. Second, we designed a structured lesson plan using a blended approach from both Gagne’s and Peyton’s instructional models. Lastly, we delineated a step-by-step strategy employing free Web-based bioinformatics module, combining it with a clinical scenario of familial hypercholesterolemia to disseminate the defined specifics of bioinformatics. Implementation of Schon’s reflective practice model indicated that the activity was stimulating for the students with favorable outcomes regarding their basic training in bioinformatics.

**Conclusions:**

To the best of our knowledge, the present lesson plan is the first that outlines an effective dissemination strategy for integrating introductory bioinformatics into a medical curriculum. Further, the lesson plan blueprint can be used to develop similar skills in workshops, continuing professional development, or continuing medical education events to introduce bioinformatics to practicing physicians.

## Introduction

### Background

The advent of high-throughput sequencing strategies and next-generation sequencing techniques has exponentially increased the output of genetic information. Although this information has contributed significantly in augmenting personalized medicine, the need for interpreting vast patient genomic datasets have made it essential for clinicians and medicine practitioners to familiarize themselves with the so called “Nuts & Bolts” of bioinformatics. Hence, one can argue that in today’s times, the inculcation of basic bioinformatics concepts and strategies in a typical medical curriculum is pivotal, which is also supported by the fact that courses have been designed to introduce bioinformatics in life sciences. [1] However, it is essential to remember that although the need for bioinformatics education and training in a medical curriculum is colossal, it has to be tailored accordingly. As recently indicated by Mulder et al, although there is an extensive range of addressees who are likely recipients of bioinformatics training, each has different needs in terms of what skills or knowledge they require and at what complexity [2]. For example, someone aiming to be a bioinformatics engineer needs exhaustive knowledge of prevailing algorithms, how they work, how to critically evaluate them, and how to translate the results. In comparison, a clinician as a bioinformatics user would need a basic level of understanding of the methods with emphasis on the interpretation of the outputs, specifically in relation to the discipline of genetics.

In medical education, although emphasis is on dissemination of knowledge pertaining to various concepts associated with genetics, rarely any curriculum addresses the pertinent question “How this knowledge can be translated to diagnosis of genetic maladies?” for which a basic outline of a typical bioinformatics analysis in line with the central dogma of molecular biology (DNA→RNA→Protein) is required. However, first, one needs to identify how “bioinformatics’ knowledge” can be translated to facilitate diagnosis specifically from the perspective of a clinician. Specifically, genetics and bioinformatics can be useful for the following 3 separate disease categories: monogenic or chromosomal disorders, such as phenylketonuria, sickle cell anemia, neurofibromatosis, or downs syndrome; more common disorders such as breast cancer, hemochromatosis (as a cause of liver disease) and cardiomyopathy for which a substantial subset of individuals have a monogenic cause or where single gene mutations can, in some families, cause the disorder; and wide spread disorders such as diabetes, hypertension, hypercholesterolemia, cancer and cardiovascular disease, which are multifactorial disorders in which multiple genes interact with one another and the environment to contribute to the cause or condition severity.

For all 3 categories, the strategy will essentially involve identifying aberrant gene sequence compared with the normal variant(s). The next step would involve assessing the severity of the aberration by translating its effect at the functional level or protein expression. Therefore, introductory bioinformatics in a typical medical curriculum needs to focus on the following 2 specifics: comparison of nucleotide sequences and prediction of how a mutation affects the structure-function of the protein it translates.

It is possible to impart knowledge of these in bioinformatics workshops. Continuing education workshops in bioinformatics has been shown to positively impact research and careers [3,4]. However, as the popularity of medical spiral curricula [5] has gained momentum, bioinformatics needs to be integrated into medical curricula, a requisite that was also highlighted by the Global Organization for Bioinformatics Learning, Education and Training [6,7]. Further, because many medical schools admit students with a high school degree (especially medical schools in the Middle East and North Africa region) who have little conceptual knowledge about sequence databases and *in silico* analysis of gene or protein sequences, education policies to examine strategies to promote the design of integrated courses, particularly bioinformatics, specifically designed to capture the expectations of medical education in the millennial era by smoothening the learning process keeping in mind the transition of students from high school to university [8] is required.

**Figure 1:**
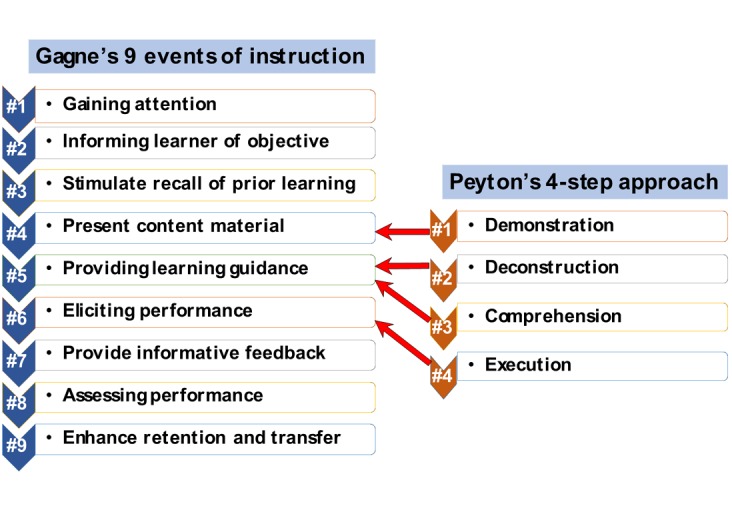
Description of the blended lesson plan.

This study addresses this need, wherein a 4-hour introductory bioinformatics lesson plan was designed and implemented in an undergraduate medical curriculum. Further, because bioinformatics is not currently integrated in typical medical curricula (even those that follow the spiral model), it is imperative that such lesson plans consider the conditions under which learning occurs and the learning goals are attained. With this in mind, the established instructional design strategies employed in medical education—Gagne’s 9 events of instruction—were followed with further contributions from Peyton’s four-step approach, (Figure 1). The figure depicts both Gagne’s 9 events of instruction and Peyton’s 4-step approach, including the steps in Gagne’s 9 events of instruction that blend inputs from the principles of Peyton’s 4-step approach (indicated by the red arrows) [9-13].

**Figure 2:**
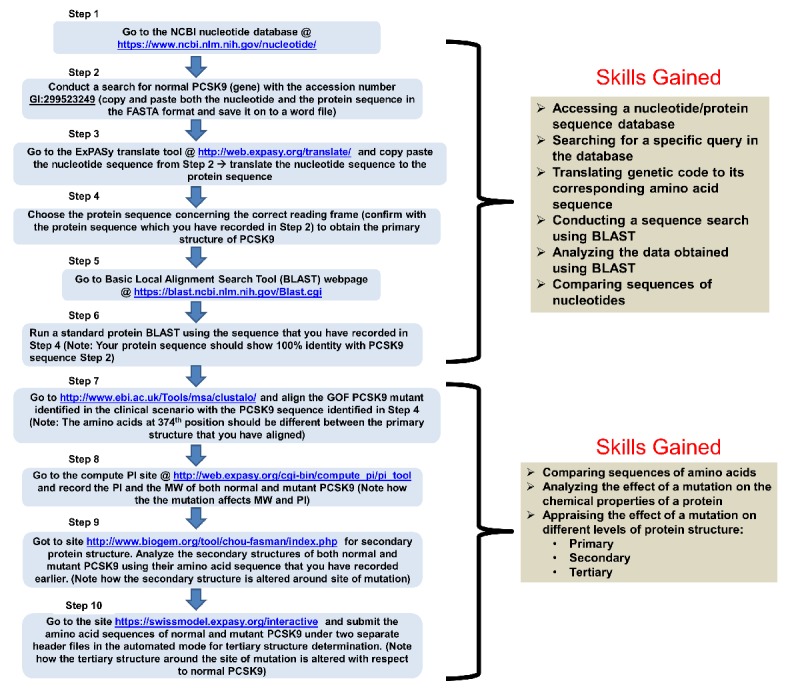
The sequential lesson plan steps. NCBI: National Center for Biotechnology Information; BLAST: Basic Local Alignment Search Tool; GOF: gain of function mutation; PI: isoelectric point, MW: molecular weight.

Over the last decade, medical teaching approaches have undergone significant transformation. Instead of conformist programs with traditional teaching approaches, most medical schools today have developed a “reformed” medical program that includes alternative and new instructional methods, such as small group problem-based learning (PBL) [14], e-learning, or case-based work for small working groups, the use of which has been found to stimulate peer-assisted [15] and self-directed learning [16]. Therefore, the best-strategy for the dissemination of the bioinformatics learning objectives for an undergraduate medical student cohort in this lesson plan was a problem-based learning approach that promoted bioinformatics self-learning [17]. Further, rather than adopting models from other contexts that might not be relevant, because the lesson plan was designed for local effectiveness [18], a familial hypercholesterolemia clinical scenario was used because this is a common genetic disorder in the Middle East [19]. However, the lesson plan outline (Figure 2) can be tailored to any clinical scenario based on the clinical needs in specific regions. The flowchart depicts the individual lesson plan steps including the Web-based software modules implemented in the bioinformatics lesson in the first year Molecular Biology and Principles of Genetics course (all software modules used in the lesson are accessible free of cost through different Web-based servers). The skills gained in each lesson plan step are indicated in the shaded boxes. The blended approach was implemented using sequential steps.

### Lesson Plan Implementation Setting

The lesson plan was successfully implemented in an undergraduate medical education (UME) first year Molecular Biology and Principles of Genetics course at Mohammed Bin Rashid University of Medicine and Health Sciences (MBRU) Dubai, UAE (a nonprofit organization managed in collaboration with Queens University of Belfast, UK), for which the entire cohort of 54 students was registered.

The designed lesson plan employed open-source Web-based bioinformatics modules [20-22]. Use of such modules will promote the implementation of this lesson-plan in any UME course in any medical school that has access to basic computer and internet resources. The plan was founded on Gagne’s model of instructional design [9], which is focused on the “information processing model” of psychological events that occur when adults are presented with various stimuli as well as learning outcomes and how to arrange specific instructional events to achieve those outcomes. Further, specific steps from Gagne’s model of instructional design were blended with Peyton’s 4-step instructional design approach, which, in recent times, has gained recognition as a suitable strategy for the augmentation of clinical or technical skills in UME [10].

## Methods

### Prerequisites

All medical students attending the lesson plan implementation session were able to use a desktop computer and had successfully completed a basic biochemistry course in their previous semester; therefore, they were all familiar with the specific concepts needed to successfully follow the lesson plan. As groundwork for the session, prior to the bioinformatics session, the students were also asked to review the following concepts: genetic codes and mutations and different protein structure levels. With these prerequisites, the students were able to perform the basic bioinformatics analyses in the lesson plan. The individual lesson plan steps were as follows and the allocated times for each step are shown in Table 1.

#### Step 1: Gaining Attention

To engage the students prior to the lesson, the instructor attracted their attention using an unexpected auditory stimulus; for example, striking the microphone to generate a crackling sound and a Maieutic technique to stimulate cooperative argumentative dialogue using a thought-provoking question “How would you explain bioinformatics to your grandmother?” A short motivational YouTube video by Spencer Hall on bioinformatics was shown (Bioinformatics: A way to decipher DNA and cure life’s deadliest diseases) [23]. This technique concurrently addressed visual, auditory, and kinesthetic learning styles [24].

#### Step 2: Informing Learners of the Objectives

Immediately following the video, the lesson objectives and the achievable lesson expectations were given to prepare the students for the learning process; for example, the predetermined objectives for this session are as follows:

Upon completion of this session, you should be able to compare gene sequences; design a strategy to translate a nucleotide sequence into its corresponding amino acid sequence; translate the effect of a genetic mutation to different protein structure levels.

These objectives were reviewed with the students to ensure they were aware of the rationale behind the lesson plan organization and the sequence of the objectives.

**Table 1:** Lesson Plan Steps based on Gagnes’s Instructional Model Blended with Peyton’s Approach

Step	Key event (allocated time)	Major happenings
1	Gaining attention (10 min)	Instructor attracts the attention of the students using suitable audio and visual stimuli Instructor encourages cooperative argumentative dialogue using thought-provoking questions
2	Informing learner of the objective (15 min)	Instructor presents the learning objectives for the session on Bioinformatics analysis Upon completion of this session you should be able to: Compare gene sequences Design a strategy to translate a nucleotide sequence into its corresponding amino acid sequence Translate the effect of a genetic mutation for different protein structure levels
3	Stimulate recall of prior learning (20 min)	Students participate in an *in silico* session in which they review different bioinformatics strategies and tools to identifying aberrant genes and single nucleotide polymorphisms
4	Present content material (40 min)	Instructor presents a scenario (refer to text) at the conclusion of which questions are asked as to which bioinformatics technique needed to be applied. Students attempt to individually answer the questions The instructor then demonstrates the individual steps in a bioinformatics analysis (using the presented scenario)
5	Providing learning guidance (10 min)	Instructor elaborates on the rationale of the steps Specific dos and don’ts are also addressed Queries stemming from the discussion of the steps with the students are also addressed by the instructor
6	Eliciting performance (40 min)	Students perform a self-assessment exercise (refer to text)
7	Provide informative feedback (25 min)	Pendleton’s feedback model is used to provide feedback for which both instructors and students appraise the activity
8	Assessing performance (50 min)	Students compare the obtained bioinformatics analysis results with the model answer uploaded on the learning management system Any ensuing questions and queries are addressed by the instructor
9	Enhance retention and transfer (30 min)	The individual lesson plan steps and the model answer are discussed side by side using both the instructor presented scenario and the self-assessment exercise Any ensuing question either from the activity or in relation to the concepts disseminated are addressed and the session is concluded

#### Step 3: Stimulate Recall of Prior Learning

Medical students are adult learners. The constructivist theory claims that adults construct their knowledge founded on connections with previous learning and experiences [25]. Therefore, the students participated in group discussion sessions in which different bioinformatics strategies for identifying diseased genes and single nucleotide polymorphism candidates were reviewed to facilitate the recall of the gene structure-function concepts. Students also referred to different Web-based bioinformatics tools such as those available on the OMIC tools website [26]. This discussion session allowed the students to revisit the genetics concepts and recognize their clinical importance with the aim of inducing knowledge creation from the pre-existing foundations. This step also informed both peer-assisted learning and multi-intelligence theories [27,28].

#### Step 4 (Blended): Present Content Material

The detailed stages involved in bioinformatics analyses were addressed using a clinical case (Textbox 1) and presented as a PowerPoint presentation with a flowchart summarizing the steps provided as a hand-out (Figure 2). To teach the actual bioinformatics procedural skills, Peyton’s 4-Step principles were integrated in this step.

Peyton’s Step 1 involved a demonstration of each of the essentials needed for a bioinformatics analysis (at a standard pace without elaborating on the steps) starting from the identification of a specific gene sequence to comparing a mutant sequence (of the identified gene) with a normal or wild-type sequence, followed by an appraisal of the effect of the mutation on the different protein structure levels. In line with Fleming’s Visual-Audio-Kinesthetic learning model [29], this step was aimed at encouraging visual-audio, philological, and relational acumens.

**Clinical Scenario used in the Lesson: Familial Hypercholesterolemia.**

**Case study**

A 29-year-old Emirati male with a body mass index of 19.5 kg m^−2^ presented with advanced tuberous xanthomata on both auricles, elbows, gluteal regions, and legs since birth.

His father and paternal and maternal grandfather had xanthelasma; however, the siblings did not. Laboratory investigations were performed on several occasions. These revealed extreme dyslipidemia with very high total cholesterol, low density lipoprotein cholesterol, triglycerides, apolipoprotein B and lipoprotein(a), and low apolipoprotein-A levels.

Repeated combinations of lipid lowering agents with cholestyramine, atorvastatin, and ezetimibe had been virtually ineffective in improving the lipid profiles, and lipid-apheresis had to be pursued.

Genetic analyses showed that the patient was homozygous for a gain of function mutation; D374Y in the PCSK9 gene; which explained the severe observed dyslipidemia. Hence, according to the Dutch diagnostic criteria familial hypercholesterolemia was confirmed in the patient.

**Deliverable or Task**

Using the provided flowchart:

Compare the sequences for the mutant and normal PCSK9 genes to identify the exon in which the aberration is located.

Identify how the mutation affects the different levels of the PCSK9 protein structure.

Identify which domain of the tertiary structure of the PCSK9 is affected by the mutation.

Compare your observations with the colleagues seated next to you. You should record the relevant observations in the figure or graphs because you will require them when you prepare your report, which should be submitted as a “.docx” file using the learning management system.

#### Step 5 (Blended): Providing Learning Guidance

This level focused on interactive learning. First, the instructor elaborated on the individual steps for the activity and clarified the rationale. Subsequently, the instructor analyzed the steps and comprehensively reiterated the individual bioinformatics analysis steps, outlined the necessary dos and don’ts, and gave some practical tips (Peyton’s Step 2). The students were encouraged to ask questions to clarify any uncertainties. This was followed by a conceptual phase in which the students clarified each of the bioinformatics analysis steps with the instructor following the directions (Peyton’s Step 3). This step stimulated philological and kinesthetic learning styles. Because students had to articulate the step-by-step analysis sequence, it allowed the instructor to assess their understanding.

#### Step 6 (Blended): Eliciting Performance

A greater proportion of the time was allocated to this step because this step gave students the opportunity to reinforce their learning through performance. Therefore, this step was equivalent to Peyton’s Step 4 in which the students (as part of a small team) attempted to complete the bioinformatics analysis using the designed scenario along the lines of Al-Waili et al [30], wherein a mutation in the PCSK9 gene leads to autosomal dominant familial hypercholesterolemia (Textbox 1). Small teams of 2-3 students collaboratively conducted the analysis with the instructor encouraging team members to follow each of the designated analysis steps and discuss the results to ensure accuracy. This step aided peer-assisted learning and created a nonthreatening positive environment for collaborative learning and the development of collective intelligence [28].

#### Step 7: Provide Informative Feedback

Pendleton’s feedback model was implemented in this step to provide informative feedback [31]. Although students were conducting their group analysis, the instructor provided individual assistance and instant feedback by visiting the individual groups. The student groups were also encouraged to clarify questions as they arose by discussing them with the entire cohort. The students provided feedback and indicated what they appreciated about the activity as well as the aspects that could be improved.

#### Step 8: Assessing Performance

This activity gave the students guided hands-on practice in using bioinformatics to investigate the effects of genetic mutations on different protein structure levels. The students also prepared a report discussing their observations (to reflect their understanding).

The instructor assessed the report based on a rubric (which was also shared with the students). The report contributed 5% to the total course assessment. Report writing can augment student “reflection” on a specific subject or scenario, which stimulates self-regulated and lifelong learning as “Reflection is a metacognitive process that creates a greater understanding of both the self and the situation so that future actions can be informed by this understanding” [32].

#### Step 9: Enhance Retention and Transfer

Following the report submission, the students assessed similar clinical scenarios as familial hypercholesterolemia (FH) using bioinformatics, such as a clinical scenario involving sickle cell disease [33], which allowed the students to make sense of the learning event.

The activity was concluded by revisiting the learning objectives and addressing any outstanding queries. Afterwards, the students were able to use bioinformatics in any research project involving genetics. These activities informed student learning through Kolb’s experiential learning cycle [34].

## Results

The formal lesson plan evaluation is still pending. However, the course coordinator and instructors followed Schon’s reflective practice model and [35] “reflected on the session” from which it was discovered that the students had enjoyed the activity and had been engaged in all lesson plan steps (Figure 2). When “reflecting on the session,” the instructors discovered that as some of the work-stations had slowed down during the session, some student groups took longer to complete the activity. Therefore, in the future, a work-station certification step has been included; in this, in liaison with the university information technology department, a designated work-station is to be certified (for carrying out the designated bioinformatics analysis) prior to the lesson.

Next, we asked the question “Was the lesson-plan effective in disseminating the desired objectives?” To address this, we reflected on the laboratory report evaluation scores. The laboratory report assessments (which were double marked) had an average score above 90% and there were no failures (standard setting was pursued using Angoff’s method [36]). Overall, 85% (46/54) of the student cohort expressed confidence in being able to apply bioinformatics in their research projects.

## Discussion

### Limitations

The aim of this lesson plan was to provide first year undergraduate medical students, who had come directly from high school and had had different curricula mixes, a preliminary understanding of the bioinformatics. Further, we suggest that this type of lesson plan be implemented in the initial years to provide students with the tools to understand the advanced bioinformatics concepts they may come across after their final year. Therefore, we identify the specific limitations of our study below, which not only highlight the importance of this lesson plan but also identify the specific domains that need to be further investigated.

1. An in-depth evaluation of the current lesson plan is needed. Therefore, a lesson plan evaluation form needs to be developed and implemented. As part of a separate study, based on Kamran et al [37], we plan to develop and validate [38] an lesson plan evaluation form and then implement it with several cohorts to ensure statistical validity.
2. Our molecular biology based lesson plan introduced bioinformatics to undergraduate medical students in the early years of the medical curriculum. This lesson plan does not develop student knowledge on next-generation sequencing data analysis because this requires extensive e-infrastructure [39], which is currently unavailable at MBRU. Also, this type of analysis is generally conducted by dedicated big data analysts (bioinformaticians) and therefore adds little to the clinical competencies needed for a safe, competent clinician.
3. Because our lesson plan is grounded in well-established instructional design models, instructors may feel that it interferes with their independence or flexibility. However, it is essential to base teaching plans on well-established instructional models to assist teachers and facilitators to stay on track [40]. Additionally, the lesson plan offers considerable flexibility to instructors on the choice of clinical scenario.

### Conclusions

Several training modules have been developed to integrate introductory bioinformatics into pharmaceutical and biomedical science curricula [41,42,43]. However, most of these have been in a modular format that requires faculty members to have a strong understanding of bioinformatics algorithmic design, data analysis strategies, and *in silico* resources and facilities that may not be available in medical schools with limited budgets and capabilities. Although there have been papers focused on pharmaceutical bioinformatics [41,42,43], the learning objectives are not the ones required for undergraduate medical students. To the best of our knowledge, the present lesson plan is the first that outlines an effective dissemination strategy for integrating introductory bioinformatics into a medical curriculum. This lesson plan employed a “blended” methodology in which Gagne’s instructional model was “blended” with Peyton’s four-step approach. In this paper, we used the lesson plan to introduce bioinformatics skills into an undergraduate medical curriculum. However, the lesson plan blueprint can be used to develop similar skills in workshops, Continuing Professional Development, or Continuing Medical Education events to introduce bioinformatics to practicing physicians. Also, the lesson plan has considerable flexibility for the teaching of introductory bioinformatics analysis with only basic computing facilities, which could be beneficial for medical schools that have small operational budgets.

## Abbreviations

FH: familial hypercholesterolemia
MBRU: Mohammed Bin Rashid University of Medicine and Health Sciences
UME: undergraduate medical education
